# Self-assembling Fmoc dipeptide hydrogel for *in situ *3D cell culturing

**DOI:** 10.1186/1472-6750-7-88

**Published:** 2007-12-10

**Authors:** Thomas Liebmann, Susanna Rydholm, Victor Akpe, Hjalmar Brismar

**Affiliations:** 1 Cell Physics, Department of Applied Physics, Royal Institute of Technology, S-106 91 Stockholm, Sweden

## Abstract

**Background:**

Conventional cell culture studies have been performed on 2D surfaces, resulting in flat, extended cell growth. More relevant studies are desired to better mimic 3D *in vivo *tissue growth. Such realistic environments should be the aim of any cell growth study, requiring new methods for culturing cells *in vitro*. Cell biology is also tending toward miniaturization for increased efficiency and specificity. This paper discusses the application of a self-assembling peptide-derived hydrogel for use as a 3D cell culture scaffold at the microscale.

**Results:**

Phenylalanine derivative hydrogel formation was seen to occur in multiple dispersion media. Cells were immobilized *in situ *within microchambers designed for cell analysis. Use of the highly biocompatible hydrogel components and simplistic procedures significantly reduced the cytotoxic effects seen with alternate 3D culture materials and microstructure loading methods. Cells were easily immobilized, sustained and removed from microchambers. Differences in growth morphology were seen in the cultured cells, owing to the 3-dimentional character of the gel structure. Degradation improved the removal of hydrogel from the microstructures, permitting reuse of the analysis platforms.

**Conclusion:**

Self-assembling diphenylalanine derivative hydrogel provided a method to dramatically reduce the typical difficulties of microculture formation. Effective generation of patterned 3D cultures will lead to improved cell study results by better modeling *in vivo *growth environments and increasing efficiency and specificity of cell studies. Use of simplified growth scaffolds such as peptide-derived hydrogel should be seen as highly advantageous and will likely become more commonplace in cell culture methodology.

## Background

Cell culture techniques are an integral part of cell analysis. Culture methods are often not a primary concern in growth studies, but rather a means to experimental results. As more understanding of its influences is revealed, the significance of cell growth methodology is receiving considerably more attention. For example, it is increasingly common to see a shift in growth studies from 2D surface cultures to 3D suspension cultures [1-3]. This clear trend is a product of growth methodology improvements arising leading to more realistic and relevant analysis results. In order to best mimic natural cell growth, *in vitro *cultures attempt to closely model the extracellular support structure naturally occurring in tissues. *In vivo *tissue growth involves cell production of extracellular matrix material, composed primarily of various proteins. Artificially produced extracellular matrices commonly used consist of mixtures of proteins such as collagen, fibrin, elastin, fibronectins, and laminins. Such components are understood to play a role in cell anchoring and separation, intercellular communication, and even nutrient feed and waste displacement. Recent studies reveal the possibility for more simplified extracellular matrix materials.

Along with the trend of 3D growth, modern cell laboratories strive to improve culture analysis platforms through size reduction and design specificity. Miniaturization dramatically increases the efficiency of studies by allowing higher throughput studies and reducing unnecessary and excessive material use. It also allows the experimental designer to develop patterns with significantly increased complexity, making concurrent parallel studies possible. This added complexity often requires formation of well defined culture volumes. Micropatterned cell analysis platforms enable the use of microfluidics for cell treatment and analysis, providing new methods for cell study with increased possibilities and improved productivity. However, this concept has proven rather problematic with previously used artificial extracellular matrices. The use of microstructures introduces significant barriers for creating stable cell suspensions confined within small compartments. Some studies have employed rapid gel formation of a continuous laminar flow of cells in solution through microchannels [4]. Others involve pumping of a temperature sensitive sol-gel, followed by induced gel formation via temperature change [1]. The many complexities associated with gelling laminar flows and precision volume pumping can be eliminated by incorporating dipeptide derivative hydrogels. The combined use of 3D growth and culture patterning incorporates the advantages of both methods for higher efficacy and increased productivity.

Earlier studies have incorporated simplistic 9-fluorenylmethyl carbamate (Fmoc) peptide hydrogel, first introduced by Xu et al. [5], as a growth substrate, adding ease of gel formation and experimental flexibility. The earliest growth study of Fmoc peptide hydrogel presented both surface (2D) and 3D growth [6], followed almost immediately by a second publication on a similar dipeptide-derived hydrogel used in 2D culturing [7]. The simplicity of the latter Fmoc hydrogel featured by Mahler et al. is a product of two dominant features. First, the hydrogel is a superabsorbent material, primarily consisting of water; stable gel formation was consistently seen with as low as 0.5 wt% peptide derivative. Additionally, the seemingly complex structure of this fibrous matrix is entirely self-assembling. Introduction of appropriate Fmoc peptide concentrations to a suitable environment induces rapid self-assembly of hollow nanotubes. This low concentration Fmoc dipeptide solution is able to support a gel conformation because of its stable network of interweaving nanotubes, forming a rigid scaffold within the aqueous dispersion medium.

Here we present a new approach and application of self-assembling hydrogels for 3D cell cultures in various structures, including but not limited to microscale structures. The novelty is in the method of formation of the cell culture, enabling simple and rapid self-assembly of a gel matrix in a wide range of cell analysis structures. Spontaneous gel formation was used to immobilize liquid cell suspensions *in situ *by simply adding a concentrated Fmoc peptide solution to the cell dispersion. Rapid formation of the fibrous network led to hydrogel formation that could adapt to the size, shape and even complexity of the patterned container. This method eliminates the problematic loading of gels or highly viscous precursors into the designed container. With increasing need for specificity of culture patterns, whether large or microscale, a flexible method for cell culture formation will prove useful, with possibilities to adapt to a plethora of structural designs.

An additional feature of the hydrogel makes it increasingly favorable for use within complex structures. Despite the stability of the assembled hydrogel samples, multiple stimuli were seen to initiate reversing of the sample and breakdown of the hydrogel structure. Previous studies with peptide nanotubes and peptide derivative hydrogels have shown response to various types of treatment, whether changing stability or degrading entirely [8-10]. The Fmoc diphenylalanine gel demonstrated sensitivity to mechanical forces, environmental changes, and enzyme digestion. Use of one or more of these treatments enabled degradation of the gel and facilitated removal from the culture system. This can be highly favorable for systems with confined or inaccessible culture compartments. Such is often the case in microscale gel cultures, where microfluidic treatment requires compartmentalization of the immobilized cells.

## Results and Discussion

### Hydrogel Formation

**Figure 1:**
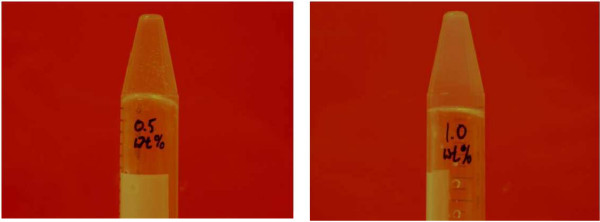
**Inversion test**. Inversion test for *in situ *assembled hydrogel samples in water. Samples contain 0.5 and 1.0 wt% peptide. The peptide source is Fmoc-Phe-Phe in DMSO (100 mg/ml).

Hydrogel samples were created from a stock gelling agent of either 25 or 100 mg/ml. Successful gelling was performed in purified MilliQ water with both stock solutions. Final Fmoc peptide concentrations ranged from 0.5 to 1.0 wt% with successful hydrogel results. Each sample within the range passed the inversion test; examples can be seen in Figure 1. The higher concentrations resulted in a gel with increased stability. Higher concentrations resulted in hydrogels that resisted deformation when mechanical force was applied. The lower concentrations were deformed with less applied stress (mixing with a pipet or vortex). Macroscale samples (1 ml) assembled stable hydrogels in less than 10 minutes, while less time was required for smaller sample volumes. Alternate dispersion media appropriate for cell studies were also tested for hydrogel assembly. For large scale samples (1–2 ml), varying media and Fmoc dipeptide concentrations were tested. The results are listed in Table 1. All of the samples prepared as culture scaffolds within microchip chambers were significantly smaller than the milliliter test volumes. Both gelling agent concentrations resulted in stable hydrogels in purified water. The smaller samples showed sufficient gelling results for dispersions 2 and 3 from Table 1. Final Fmoc peptide concentrations for *in situ *hydrogels may vary from the macroscale samples, as they are dependent on the extent of dispersion within the microchamber. All hydrogels were assembled at room temperature (~20°C) so temperature effects on hydrogel self-assembly was not addressed.

**Table 1:** Hydrogel formation results

**Dispersion Medium**	**Gelling Agent Concentration (mg/ml)**	**Final Peptide Concentration (wt%)**	**Hydrogel Assembly Result**
1. PBS 10×	100	0.5	Unstable
2. PBS 1×	100	0.5	Unstable
3. PBS 1×	100	1.0	Partial
4. PBS 1×	25	0.5	Soft Gel
5. PBS 1×	25	1.0	Firm Gel
6. MEM	25	0.5	Unstable

Result of *in situ *gel formation in multiple dispersion media and Fmoc dipeptide concentrations (macroscale). All gelling agent concentrations and final Fmoc dipeptide concentrations produced stable gels in de-ionized water.

In the case of microscale gel formation, loading a microfluidic device with a highly viscous gel can be rather complex and difficult to consistently reproduce. Some traditional gels used for cell immobilization are highly temperature sensitive and require thermal control to prevent early gel formation. The peptide-derived hydrogel presented here requires no temperature regulation and can be designed to form entirely within the structure designed for microscopy cell analysis (detail provided in methods section).

In addition to simplified handling, *in situ *formation allows for more flexible experimental design. Since gel formation is initiated within the assay structure, the precursor is an aqueous solution that can be dispensed into a wide variety of patterned platforms. In this way, the hydrogel can be confined within an assay chamber designed with high experimental specificity. This allows for more complex design possibilities without the concern of difficult gel loading. The useful feature of adapting to various confining structures was demonstrated by removing a rigid gel sample formed within a conical mold.

**Figure 2:**
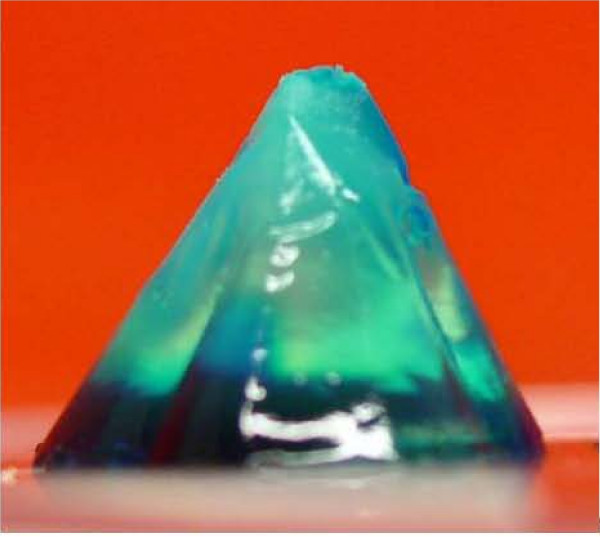
**Hydrogel mold**. Firm hydrogel formed in a conical mold. Green stain was applied to the bottom after complete gel formation, demonstrating diffusion into solid gel. Height of the sample is approximately 20 mm.

To show the absorbing character of the hydrogel, a sample was stained after the stable gel was formed. Addition of a dye to the sample after removal from the mold revealed a slow diffusion into the gel. Diffusion rates were not determined, but transport of the applied dye demonstrated the diffusion of dispersion medium. With a highly rigid structure, the shape was maintained, determined entirely by the contours of the mold. A resulting stained gel is shown in Figure 2.

**Figure 3:**
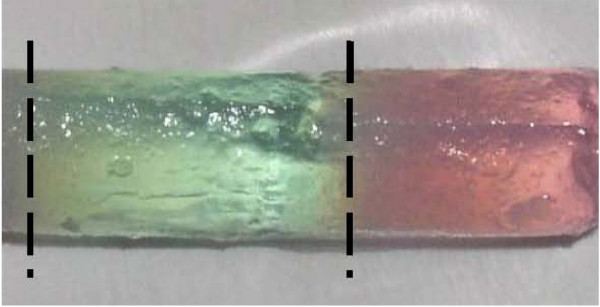
**Hydrogel layering**. Sample of hydrogel layering. Layers are distinguished by colored dye in dispersion media. Each layer interface is indicated by a dashed line.

Once the gel is created in a mold, additional layers can be formed by adding a new layer of dispersion medium and applying additional gelling agent. With formation of each layer, the assembling hydrogel binds to the previously formed layer, resulting with an adherent stack. With this method, patterned layers can be formed in a well defined volume and shape with the possibility to vary composition between layers. A sample of gel layering is presented in Figure 3. In this figure, one layer was formed upon a previously set gel layer within a cylindrical mold. The layers are distinguishable by the varied dispersion media colors, with some diffusion seen across the layer boundary. The possibility to generate layered patterns of hydrogel samples could also prove advantageous for experimental studies. The boundary condition at the layer interface can be utilized to examine cell response to environmental changes. By varying the cells suspended in each layer, the interaction between differing strains can also be observed. This opens new possibilities for 3D cell studies at a well defined interface.

### Cell Immobilization

**Figure 4:**
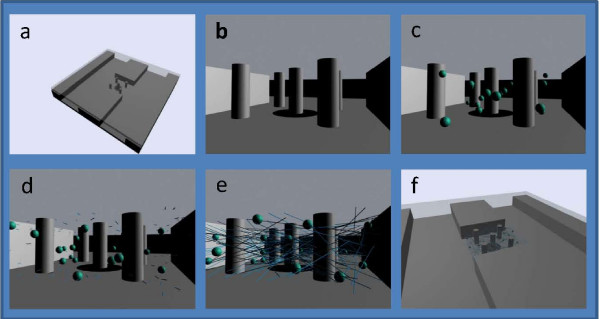
**Simulated loading of microchamber**. a) entire view of microchip system. b) close-up of empty chamber. c) after injection of aqueous cell suspension. d) spontaneous self-assembly of nanotube gel matrix. e) immobilization of cells in 3D hydrogel. f) view of cells confined solely to assay chamber.

**Figure 5:**
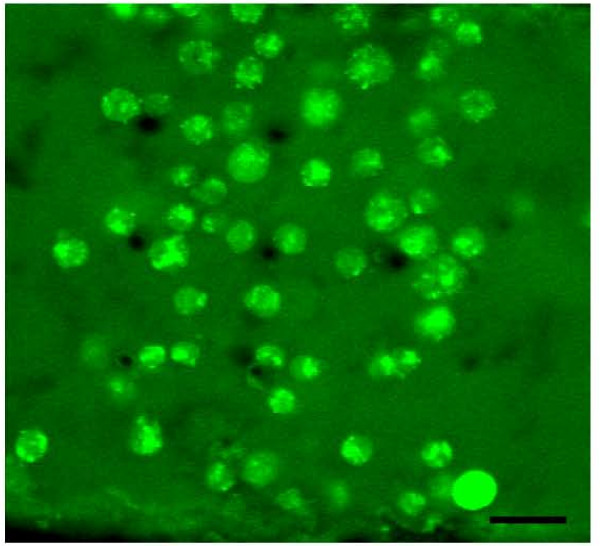
**Immobilized cells**. COS-7 cells immobilized in a 3D hydrogel within a microchamber. The scale bar represents 30 μm.

Hydrogel samples were used to immobilize cells in culture plates as well as in various microstructures. We demonstrated the ease of generating well defined 3D cell suspensions within patterned silicon microstructures. Hydrogel was formed from aqueous cell suspensions within chambers designed for microfluidic treatment and confocal analysis. The three different cell types used for suspension studies were selected because they are typically used in mammalian studies as human models; Astrocyte (two strains), MDCK and COS 7 cells were used. Figure 4 presents a simulation of the microchip loading process. A sample of cell immobilizing hydrogel formed within a microstructure is seen in Figure 5. The fluorescing cells are seen suspended within the chamber designed with 5 μm vertical pillars (seen as dark circles in the figure).

**Figure 6:**
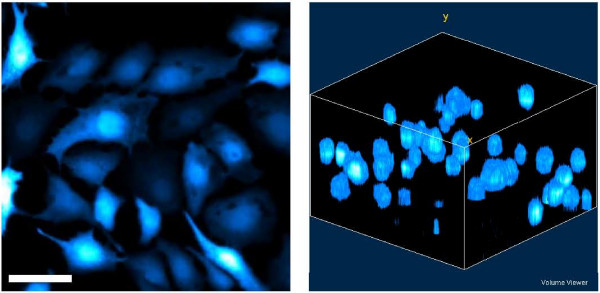
**Morphology comparison**. 2D surface growth on glass (left) and 3D growth in hydrogel (right). Scale bar in 2D image represents 50 μm. Images are of MDCK cell cultures.

Figure 6 shows a comparison of cells grown on a glass surface with a projection of cells suspended in 3D within the *in situ *assembled hydrogel. The 3D image was generated from a stack of laser confocal microscopy scans of 10 μm increments over total thickness of 160 μm. The length and width of the image scale to 230 μm each. Cells grown on a 2D substrate tend to elongate on the surface and grow significantly larger than the spherical cells suspended in hydrogel, with typical lengths of 50 μm and 10–20 μm for surface and bulk cultures, respectively. This demonstrates the rather significant morphology difference between the dimensionally different methods of cell culture.

As seen in naturally occurring human tissues, cells often grow in a 3D network. Given this spatial orientation, cells interact with both their surroundings and each other in a way fitting to their environment. Cellular function is likely to be significantly different when provided different stresses and orientation. The morphology difference demonstrated here is a closer to realistic tissue conformations. More realistic cell function, as affected by realistic cell morphology, should give rise to more relevant experimental results. For better understanding of the cell immobilization and morphology changes, the cell-hydrogel interaction should be further examined. As cell attachment is considered significant to metabolic activity and general cell function, examination of cell-hydrogel interaction would improve understanding of the cell's level of tolerance for the hydrogel environment.

**Figure 7:**
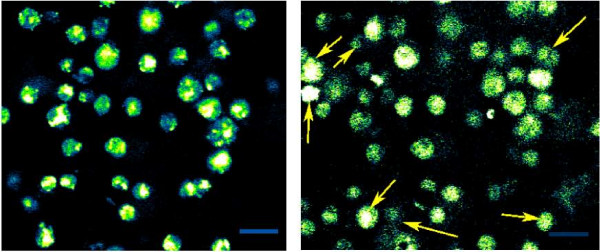
**Cell proliferation/motility**. Some migration is noticeable as COS 7 cells shift in or out of the focal plane. Most of the original cells are seen to maintain their initial position and new cells are present (indicated by arrows). Scale bar represents 25 μm. The left image is after 4 days of incubation, and the right image is after 5 days of incubation.

An initial 3D growth study demonstrating cultivation of Chondrocytes in preassembled (as opposed to *in situ *assembly) Fmoc diphenylalanine hydrogels reported that limited or no net proliferation was seen after the first three days of cultivation, while measurable growth was evident after 7 days [6]. We performed additional growth studies with all of our cell strains. Over the first few days of culturing, relatively little growth was detected (none in most test samples) while cell viability remained. Viability was verified by administering calcein-AM to the hydrogel culture. Viable cells were observable after calcein-AM uptake and internal modification to fluorescent calcein. Figure 7 presents an example of cell proliferation and/or migration in an *in situ *assembled hydrogel plug. This comparison of identical planes within the microchamber is between 4 and 5 days of incubation, for left and right images respectively. A majority of the cells remain in a fixed position, while some migration is seen with cells shifting in and out of the focal plane. There is a positive net change in cell number density shown between the images, resulting from a net flow into the focal plane and/or growth of new cells. Additional testing should be performed to differentiate the cell density change owing to migration from that of proliferation.

### Gel Reversing

**Figure 8:**
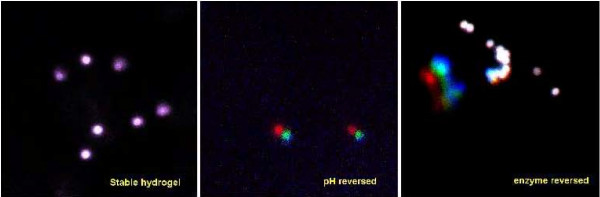
**Hydrogel Reversibility**. Time resolved confocal scan of 15 μm spheres suspended *in situ *with pH and enzyme triggered reversing. Color indicates time; red is the start time, green is at 5 minutes, blue is at 10 minutes. Lack of color differentiation indicates limited mobility.

Hydrogel samples were additionally tested for reversibility. Introduction of mechanical stress was shown to disrupt the gel network and reduce the gel stability. Sheer forces were applied by pipette or vortex mixing to degrade the gel. Once the initial gel was disrupted, a fully stable, cohesive gel was unattainable. Hydrogel formation was seen to be pH sensitive. It follows that the stability of the gel is also a condition of the environment pH. A stable sample modified from neutral pH to above pH 8.0 would no longer pass the inversion test. Further elevation to pH 9.0 was utilized to ensure extensive removal from structures (described in next paragraph). More detail of the stability was revealed with microscopic examination of the samples. Suspended microspheres (15 μm diameter) were monitored over a 10 minute period after an elevation of the pH. Hydrogel reversing was also examined via enzymatic digestion. A gel sample (5 mg/ml Fmoc dipeptide) was treated with the digestion solution (final proteinase K concentration of 10 μg/ml) and compared to a control treated with an equivalent volume of water. Some macroscale reversing effects were noted after incubation at 37°C for one hour. Inversion testing showed partial degradation of macroscale samples. Reversing was also performed in the presence of microspheres. Figure 8 shows the acquired microscopic results for both pH and enzymatic reversing. Both pH and enzyme treatment increased mobility of suspended particles.

**Figure 9:**
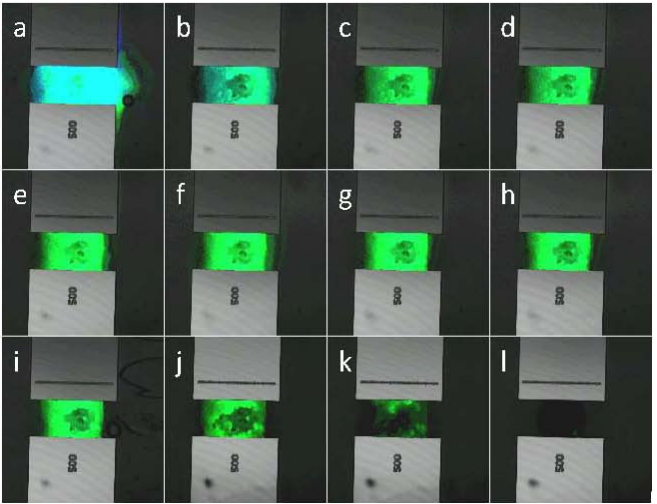
**Hydrogel Removal**. Removal of a stable hydrogel plug from a microchip assay chamber with perfusion of enzymatic removal solution. Time interval between frames is 90 seconds. Each frame is labeled chronologically, with 'a' as the initial frame. The assay chamber is 500 μm wide.

Further gel reversing was performed within microstructures via continuous pumping (specific protocol in Methods section). Active flow of the reversing solution increased breakdown of the hydrogel by combining flow-generated sheer stress, pH modification (pH 9.0 and above), and enzyme digestion. This method was sufficient for significant removal of hydrogel plugs within microstructures. Figure 9 shows confocal images of hydrogel removal from a microchip. Nearly all traces of the gel have vanished after a 16.5 minute perfusion of the reversing solution. Extensive removal from micropatterned culture chambers was less attainable with preassembled hydrogels as they were more difficult to confine exclusively to the microchamber. Any overfilling limited the ability to remove stable gel from the microstructures. Complete removal of other 3D matrix gel was extremely difficult and reuse of the microstructures was not feasible.

## Conclusion

Introduction of Fmoc peptide hydrogels to cell culture methodology has shown potential to both simplify experimental procedures and increase protocol flexibility. *In situ *hydrogel formation eliminates the complexities associated with gel handling. With gelling, the samples become more difficult to handle and apply to the actual observation/analysis platform. The peptide-derivative hydrogel simplified both handling and loading of the gel to the microstructures. With easier gel formation, it is plausible to design and construct more complex culture systems. This opens the door to new possibilities and new approaches to cell biology.

One intriguing possibility that this hydrogel enables is the study of cells at an interface. With the ability to easily control the cell type, cell density and composition of each given hydrogel layer, interactions can be studied at a well defined interface. This culture method allows one to study cellular interaction in a controlled manner that can be adapted to a variety of scales and designs.

Use of the hydrogel as a cell culture medium showed many additional benefits. Living cells suspended within the dispersion medium were easily immobilized with addition of the gelling agent. Immobilization of the cells allows for easy monitoring over extended period of time. Microscopic studies demonstrated that the immobilized cells were not restricted from migration or possible proliferation, while maintaining high viability.

Another significant benefit of the hydrogel as a culture method is the improved relevance. The goal of mimicking *in vivo *cell growth required a more realistic spatial growth arrangement than conventional 2D surface growth. Immobilization of suspended cells allows for a 3D dispersion of cells throughout the gel. This arrangement more closely models the *in vivo *growth of cells seen in tissues. Microcopy also revealed that cells suspended in a hydrogel sample tend to adopt a 3D structure, rather than the elongated conformation seen in surface cultures. The support provided by a 3D gel results in more spherical cell growth. This may have significant implications in cell studies as more relevant and realistic results are desired.

With a trend toward miniaturization of cell studies, microstructures become more common culture platforms. As micropatterning can carry a significant expense, the ability to reuse the microstructures can be economically preferable. Traditional gels used in 3D cell culture can be extremely difficult to remove from microstructures due to the increased surface interactions. Hydrogel reversibility by means of applied force, environmental change, or enzymatic digestion facilitates breakdown of the rigid gel structure. Removal of the hydrogel culture can then allow for repeated experiments within a single device. Hydrogel reversing was observed, but could be improved with further studies. Increased specificity of enzyme activity could dramatically increase the rate and extent of Fmoc peptide digestion. With improved reversal, total removal could be achieved with non-intrusive methods, possibly facilitating downstream recovery of cells for additional testing.

## Methods

### Gel Formation

The self-assembling hydrogel samples were made by first creating a gelling agent. Lyophilized Fmoc-Phe-Phe dipeptide (Bachem) was weighed and dissolved in dimethyl sulfoxide (DMSO). Deviating from the preliminary methods provided by Mahler et al., DMSO was selected to replace 1,1,1,3,3,3-hexafluoro-2-propanol because it is less detrimental to cell viability. The gelling agent was used in concentrations of 25 and 100 mg/ml. A small stock solution was created to run experiments for each day so as to avoid any pre-aggregation of dipeptide derivatives. The gelling agent was applied to the desired dispersion medium by pipette addition to a final concentration of either 5 or 10 mg/ml; the higher concentration corresponded to increased gel stability. The gel was created *in situ *within the desired analysis structure. All structures were fabricated via silicon etching/masking procedures by the Microsystems Technology Group in the School of Electrical Engineering, Royal Institute of Technology, Sweden. Transparent glass cover slides were bonded to the etched surface to create channels and chambers for microscopic observation.

Layer formation was performed by repetition of the gel forming procedure. The initial layer was first generated in a generic mold and allowed to stabilize. Upon stabile hydrogel formation, the additional dispersion media was added to the mold, forming a fluid layer above the stable hydrogel. An appropriate amount of gelling agent was directly added to the fluid layer. Layers were only formed in purified water, but it can be assumed that all successful dispersion media can also be used in layer formation. We only examined the use of 25 mg/ml gelling agents for a final hydrogel concentration of 10 mg/ml. Diffusion of dispersion medium in layers was observed by adding a colored dye in one layer of hydrogel. For general diffusion observation, a drop of dye was applied to a stable get and allowed to diffuse into the sample.

### Gel Reversing

**Figure 10:**
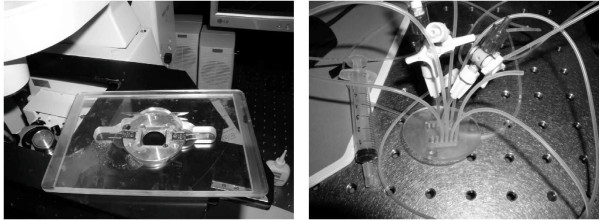
**Microchip platform and Perfusion System**. Left image is the microchip platform designed for a confocal microscope stage. Right image is the tubing configuration for continuous perfusion that was performed via a syringe pump and the tubing configuration presented here. Holes in cover-plate (right) coincide with inlet and outlet holes in the microchannels.

Mechanical reversing was performed by applying sheer forces to a stable gel. Large samples were destabilized by vortex or pipette mixing. Environmental pH changes were also used to reverse assembled gel stability. NaOH (0.5M) was added to elevate the matrix solution above pH 9. Enzyme reversing was performed with proteinase K (Sigma-Aldrich). Reversing in microstructures was performed by actively applying a flow of reversing solution: 0.05 mg/ml proteinase K, 1.0 wt% SDS, pH between 9 and 10, and temperature control at 37°C. Microchambers were all flanked by parallel channels (Figure 4) where the enzyme reversing solution was perfused. Precision pumps were fitted to drive the solution from a syringe, through flexible tubing, into the inlets of the parallel microchannels, and out through the outlets. The microchip stage and perfusion tubing setup is shown in Figure 10.

### Detecting Gel Transitions

To confirm gel formation, two protocols were employed. The first was a macroscopic observation of the gel stability. In the presence of a rigid conformation, the hydrogel should be sufficiently stable to undergo and inversion test without conformational changes. Inversion testing was performed on 2 ml samples in a 15 ml Falcon Tube. After appropriate stabilization time, the sample was inverted and monitored for deformation. A sample undergoing incomplete gel transition shows deformation in the form of flow toward the lid (lowest point of the inverted tube). A complete and stable hydrogel transition was characterized by an inversion test resulting in zero visible deformation. A successful inversion test confirmed stable macroscopic hydrogel formation.

The second protocol utilized to verify gel stability required observation at the microscopic level. Optically distinguishable foreign material was added to the gel and monitored by confocal microscopy. The 'impurity' behavior was then used to infer hydrogel characteristics. Fluorescent microspheres (15 μm Focal Check microspheres, Molecular Probes) were suspended in the dispersion medium of choice just prior to addition of the gelling agent. The microspheres were then induced with 488 nm laser excitation on a Zeiss LSM 5 Pascal confocal microscope over a 10 minute detection time interval. Confocal tracking of the microspheres presented the level of stability of the hydrogel matrix in the form of sphere immobility. Immobilization of the suspended microspheres over an extended time period was a sufficient indication of a stable hydrogel matrix.

Stable hydrogel samples were monitored for sensitivity to multiple reversing methods. The above gel transition methods were utilized to demonstrate reverse transitions from stable a stable hydrogel matrix to a less stable liquid solution. If a stable hydrogel sample can be modified such that it no longer satisfies the discussed requirements for a stable gel, the sample has reverted from a gel to a liquid phase solution. More explicitly, a sample that fails the inversion test or shows a dramatic increase in microscopic movement upon modification is no longer considered a stable hydrogel.

### Cell Inclusion

The cell lines examined in hydrogel cultures include COS-7, MDCK, and two strains of Astrocytes (CTX TNA2 and DI TNC1), all obtained from ECACC. Each cell line was cultured according to the ECACC recommendations. Astrocytes were grown to confluency in a liquid culture of MEM (Gibco) and the remaining cell lines were grown in DMEM (Sigma). Cells were grown with an addition of 10% fetal bovine serum (Gibco), 1.0% penicillin/streptomycin (Sigma) and 1.0% L-glutamine (Sigma). MDCK cells were grown with an additional treatment of 1.0% non-essential amino acids (Sigma). Incubation was performed at 37°C with 5% CO2 and 100% humidity. The cells were harvested from the culturing petri dishes by trypsinization. The harvested cells were spun down to a pellet by centrifugation for 5 minutes at 1700 rpm. Cells were then washed twice before a final spin down. Staining treatments and washing were performed with a standard phosphate buffered saline solution (1× PBS). The final cell pellet was then immobilized in the peptide derivative extracellular matrix by first suspending in PBS and adding the gelling agent to initiate hydrogel self-assembly. A typical cell number density used was between 5 and 12 million cells/ml of final gel volume. Nutrients were supplied to the immobilized cell suspensions by perfusion as soon as the hydrogel was stabilized. For long-term culturing, the microstructures were removed from the microchip platform and submerged in culture media under 37°C. Cells were viewed by staining with 1.0 μM calcein-AM (Invitrogen). If the cells were to be stained before *in situ *culture formation, calcein-AM was added to the dispersion medium. For *in situ *staining within the microchamber, calcein-AM was added to a perfusion solution of growth medium and perfused through the microchannels. Cells were maintained in stain solution for 30 minutes to 1 hour at 37°C before confocal observation.

## Authors' contributions

TL was the primary researcher for this project. He also designed the project and wrote the manuscript. In addition to assisting with cell handling, SR provided instruction and guidance for inclusion of the microsystems and microfluidics. VA provided general assistance and guidance in chemical synthesis. HB was the guiding supervisor for both the experimental work and the manuscript writing. All authors have read and approved the manuscript.

## References

[B1] Frisk T, Rydholm S, Andersson H, Stemme G, Brismar H (2005). A Concept For Miniaturized 3-D Cell Culture Using an Extracellular Matrix Gel. Electrophoresis.

[B2] Paguirigan A, Beebe DJ (2006). Gelatin Based Microfluidic Devices for Cell Culture. Lab on a Chip.

[B3] Albrecht DR, Underhill DH, Wassermann TB, Sah RL, Bhatia SN (2006). Probing the Role of Multicellular Organization in Three-Dimensional Microenvironments. Nature Methods.

[B4] Minseok KS, Yeon JH, Park JK (2007). A Microfluidic Platform for 3-Dimensional Cell Culture and Cell-Based Assays. Biomedical Microdevices.

[B5] Zhang Y, Gu H, Yang Z, Xu (2003). Supramolecular Hydrogels Respond to Ligand-Receptor Interaction. JACS.

[B6] Jayawarna V, Ali M, Jowitt TA, Miller AF, Saiani A, Gough JE, Ulijn RV (2006). Nanostructured Hydrogels for Three-Dimensional Cell Culture Through Self-Assembly of Fluorenylmethoxycarbonyl-Dipeptides. Advanced Materials.

[B7] Mahler A, Reches M, Rechter M, Cohen S, Gazit E (2006). Rigid, Self-Assembled Hydrogel Composed of a Modified Aromatic Dipeptide. Advanced Materials.

[B8] Qiu Y, Park K (2001). Environment-Sensitive Hydrogels for Drug Delivery. Advanced Drug Delivery Reviews.

[B9] Toledano S, Williams RJ, Jayawarna V, Ulijn R (2006). Enzyme-Triggered Self-Assembly of Peptide Hydrogels via Reversed Hydrolysis. JACS.

[B10] Reches M, Gazit E (2003). Casting Metal Nanowires with Discrete Self-Assembled Peptide Nanotubes. Science.

